# A Population-Based Study Examining Hepatitis B Virus Infection and Immunization Rates in Northwest China

**DOI:** 10.1371/journal.pone.0097474

**Published:** 2014-05-15

**Authors:** Zhaohua Ji, Tingcai Wang, Zhongjun Shao, Dahong Huang, Anhui Wang, Zhiwen Guo, Yong Long, Lei Zhang, Haixia Su, Qi Zhang, Yongping Yan, Daiming Fan

**Affiliations:** 1 Department of Epidemiology and the Ministry of Education Key Lab of Hazard Assessment and Control in Special Operational Environment, School of Public Health, Fourth Military Medical University, Xi'an, China; 2 Wuwei municipal Center for Disease Control and Prevention, Gansu, China; 3 Xijing Hospital of Digestive Diseases, Fourth Military Medical University, Xi'an, China; Centers for Disease Control and Prevention, United States of America

## Abstract

**Background and Aim:**

Current baseline data regarding the prevalence of hepatitis B virus (HBV) infections and the immune status in hyperendemic areas is necessary in evaluating the effectiveness of ongoing HBV prevention and control programs in northwest China. This study aims to determine the prevalence of chronic HBV infections, past exposure rates, and immune response profiles in Wuwei City, northwest China in 2010.

**Methods:**

Cross-sectional household survey representative of the Wuwei City population. 28,579 participants were interviewed in the seroepidemiological survey ≥1 year of age. House to house screening was conducted using a standard questionnaire. All serum samples were screened by enzyme-linked immunoassays for the presence of hepatitis B surface antigen, antibodies against HBV surface antigen, and antibodies to the hepatitis B core antigen.

**Results:**

Among individuals ≥1 year of age, 7.2% (95%CI: 6.3–8.1%) had chronic HBV infections, 43.9% (CI: 40.4–47.4%) had been exposed to HBV, and 23.49% (CI: 21.6–25.3%) had vaccine-induced immunity. Multi-factor weighted logistic regression analysis showed that having household contact with HBV carriers (OR = 2.6, 95%CI: 2.3–3.0) and beauty treatments in public places (OR = 1.2, 95%CI: 1.1–1.3) were the risk factors of HBV infection in whole population. Having household contact with HBV carriers (OR = 3.8, 95% CI: 2.2–6.5) and lack of hepatitis vaccination (OR = 2.0, 95% CI: 1.4–3.3) were the risk factors for HBV infection in children aged 1–14 years.

**Conclusions:**

Hepatitis B infection remains a serious public health problem in northwest China. Having household contact with HBV carriers and beauty treatments in public places represented HBV infection risk factors. Hepatitis B vaccine immunization strategies need further improvement, particularly by targeting the immunization of rural migrant workers.

## Introduction

Hepatitis B virus (HBV) infections represent the leading cause of illness and death in China. Every year, an estimated 300,000 persons in China die from HBV-related liver cancer or cirrhosis, accounting for 37–50% of HBV-related deaths worldwide[1], [2]. This disease results in tremendous economic and healthcare burdens. The Chinese National Hepatitis Seroepidemiological Survey found that the prevalence of hepatitis B surface antigen (HBsAg) in individuals aged 1–59 years was 7.2%, and that the hepatitis B epidemic in China shifted from high to intermediate endemicity following implementation of effective nationwide vaccination programs[3]. However, the incidence of HBV in western China remains high with a prevalence of HBsAg of 8.2% in 2006[3], which was significantly higher than rates observed in eastern China and most Western countries[3], [4].

In order to prevent and control HBV transmission, a comprehensive prevention strategy was initiated in 1992 that included universal vaccination of infants; screening of all pregnant women for HBV, administration of postexposure prophylaxis to infants born to HBsAg positive women, catch-up vaccination of children and adolescents, and vaccination of adults who were at increased risk of infection[5]. However, many newborns, children, and adolescents were not vaccinated due to high vaccination costs, especially in economically underdeveloped regions in western China[6], [7]. The hepatitis B immunization coverage was 33.5% among children born between 1992–2005 in western China, which was significantly lower than other regions of China[7].

The reported incidence rate of HBV in Wuwei City increased from 571/100,000 cases in 2005 to 742/100,000 cases in 2008, which was the highest in China and 8 times higher than the national average according to National Disease Supervision Information Management System (NDSIMS) which is a direct reporting network system for notifiable infectious diseases in China[8]. However, analysis of surveillance data has not always resulted in similar conclusions. For example, one study indicated that the incident rate of HBV infections was lower than the reported incident rate described by the NDSIMS and that the infection rates did not increase between 2005–2007 in China[9], [10]. Other studies that focused on high-risk groups showed different HBV prevalence[11]–[15]. Therefore, it is unclear whether the prevalence of HBV infections in western China has decreased in recent years because population-based studies have been rather sparse. This report describes a population-based study designed to determine the HBV infection baseline rates in Wuwei City and to estimate HBV infection and exposure rates in addition to characterizing the immune status in the population.

## Methods

### Study population and sampling methods

**Figure 1:**
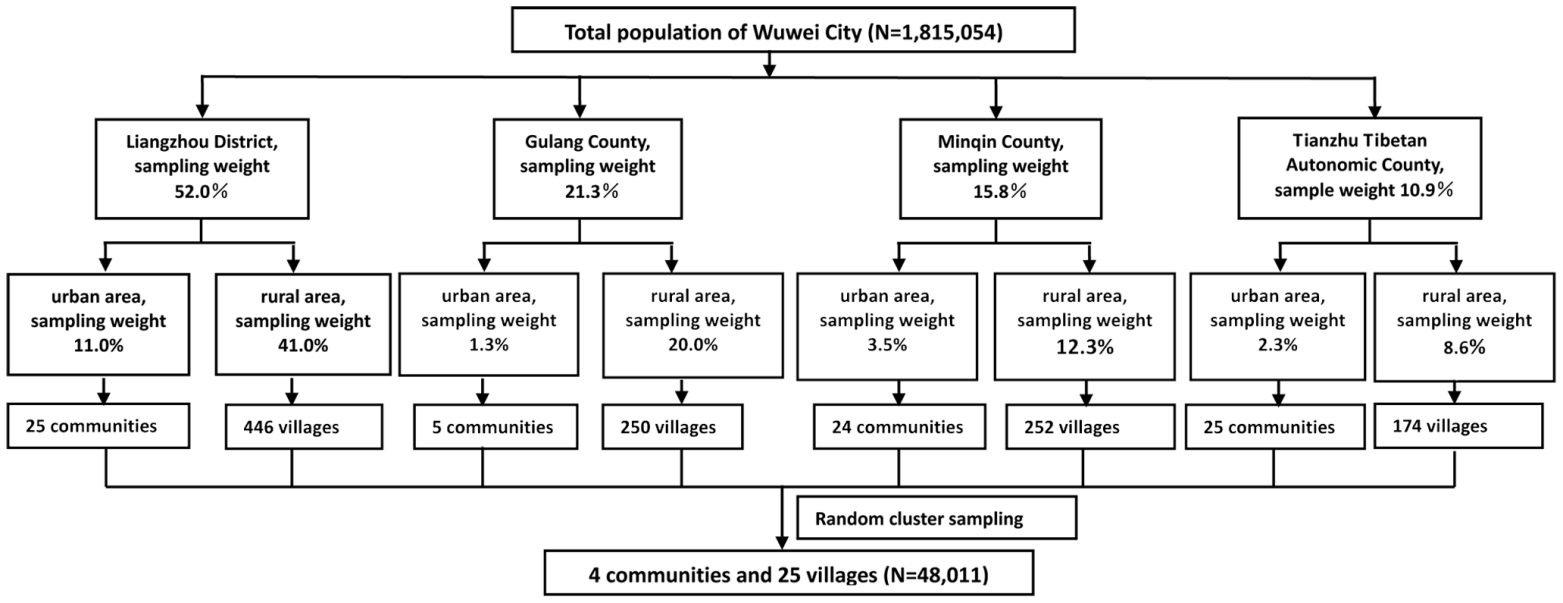
Flow chart summarizing the sampling method.

The target population consisted of local residents from Wuwei City (Gansu province). Participants were selected by the Fourth Military Medical University (FMMU) and are representative of the Wuwei City population (1.81 million). There were 4 administrative divisions: Liangzhou District, Gulang County, Minqin County, and Tianzhu Tibetan Autonomic County. Each region consisted of urban and rural populations. The Wuwei population was divided into 8 sections according to 4 administrative divisions characteristic with urban or rural populations. A stratified random cluster sampling was used according to the proportions of each section (Fig 1). 48,011 persons were selected from 4 communities and 25 villages.

### Approach

House to house investigations were conducted by trained, local centers of disease control (CDC) staff members between June 2010 and September 2010. Face-to-face interviews with study subjects or their parents or guardians (if study subjects were <15 years of age) using a standard questionnaire were used to capture demographic information including gender, age, education, occupation, ethnicity, marital status, household contacts with HBV carriers, immunization history, and history of invasive medical procedures. For children <15-years of age, immunization status was determined by asking parents or guardians about the child's immunization records. If at least one vaccine dose was administered, respective individuals were defined as ‘vaccinated’. If the vaccination certificate was unavailable, the vaccination status was recorded as ‘unknown’. Because there are no personal immunization records for adults, the immunization information for adults was based on memory (vaccinated, unvaccinated, unknown). Education levels of the study participants (and their respective occupations) was determined and based on the Chinese social classification criteria. Four field pilots were conducted before the survey. Appropriate visiting times were considered to ensure high response rates. The accuracy of 5% of the questionnaires was verified via telephone interviews conducted by FMMU staff members and the information was 99.64% consistent.

### Laboratory testing

Serum was collected from blood samples (5 ml for individuals >6 years of age and 2 ml for children ≤5 years old) and stored at −80°C. All serum samples were screened by enzyme-linked immunoassays (ELISA) for the presence of HBsAg, antibodies against HBV surface antigen (anti-HBs), and antibodies to the hepatitis B core antigen (anti-HBc) (Wantai Laboratory System, Beijing). Specimens that tested negative were considered negative and not tested further. Specimens testing positive for HBsAg were re-tested by EIA (Abbott Laboratories, Chicago, Illinois), and samples that were double positive were considered to be confirmed positive. 300 specimens were randomly selected and retested using the Abbott EIA (Abbott Laboratories) for the detection of anti-HBc and anti-HBs. The sensitivity and specificity were 100% and 99.4% for anti-HBs and 98.7% and 97.2% for anti-HBc, respectively.

### Definition of HBV infection, exposure, and vaccination status

Chronic HBV infection was defined by a positive serum HBsAg result. Exposure to HBV was defined by a positive anti-HBc result, which indicates either past or present infection. Individuals positive for anti-HBs and negative for anti-HBc were considered to have vaccine-induced immunity[4].

### Data analysis

All data were entered twice into an EpiData 3.1 software database and checked for consistency. After verifying accuracy, the data were analyzed at FMMU using SAS 9.2 (SAS Institute, Cary, NC). The Taylor Series Linearization method was used to account for the complex sampling design and provided the exact estimate of the standard errors. The weighted prevalence rates of HBV markers were calculated based on gender, age, education level, occupation, ethnicity, and other selected determinants. Weights were created to account for the different selection probabilities of persons across sub-districts, communities and villages, age, and gender. The weight for each person *i* was expressed as follows: W*_kji_* = P*_k_*×*P_j|k_*×P*_i_*, where P*_k_* equals the reciprocal of the sampling weights of sub-districts *k*, P*_j|k_* equals the reciprocal of the conditional inclusion probability of community/village *j* within the selected sub-districts *k*, and P*_i_* is an adjustment factor for person *i* according to the age and gender composition of the 2010 census population of Wuwei City obtained from the local statistics bureau. The prevalence rates of HBV seromarkers include the point estimates and their estimated 95% confidence intervals (95% CI). 95% CIs were compared for HBV marker prevalence of each variable; 95% CI which did not overlap were considered as statistically significant P = 0.05[3].

Univariate and weighted multiple logistic regression analyses for HBV chronic infections, exposure to HBV, and vaccine-induced immunity were analyzed to determine the unadjusted and adjusted odds ratios (OR) and 95% confidential intervals (95%CIs) of selected determinant risk factors associated with HBV exposure/infection among the population studied. Multivariate analysis provided useful insight into risk factors for both adults and children in addition to showing a clear effect of the selected risk factors.

### Ethical considerations

The study protocol was approved by the Ethics Committee of FMMU and consent was obtained from all participants or parents/guardians. All participants or parents/guardians signed the informed consent and provided their written consent on the first page of the questionnaire in this study.

## Results

### 1. Study population and infection rates

Study participants (n = 48,011, ≥1 year of age) were enrolled and 28,579 (59.5%) were interviewed. 19,432 (40.5%) were excluded because they worked out of the town (designated rural migrant workers) during the period of investigation (14,950,31.1%) and refused to participate (4,482,9.3%). Most of the rural migrant workers were 16–40 years of age (13,241, 88.6%) and male (9,275, 62.0%). There were 28,044 (98.1%) blood samples obtained from the 28,579 participants interviewed. Among individuals ≥1 year of age, 7.2% (CI: 6.3%–8.1%) had chronic HBV infections, 43.9% (CI: 40.4–47.4%) had been exposed to HBV, and 23.49% (CI: 21.6–25.3%) tested positive for serum anti-HBs and negative for anti-HBc suggesting this group had received an HBV vaccine (Table 1, Table 2 and Table 3). The weighted prevalence rates adjusted to represent the Wuwei City population ages 1–59 years of age were 7.4% for HBsAg, 53.1% for anti-HBs, and 41.2% for anti-HBc.

**Table 1:** Prevalence and Determinants of Chronic Hepatitis B Virus Infections (Positive HBsAg) Among Persons ≥1 Year of Age in Wuwei City, China.

Variables	Investigated, n	Prevalence of HBsAg (95%CI),%	Unadjusted OR (95%CI)	Adjusted OR (95%CI)		
				Age ≥1 y^e^	Age 1–14 y^e^	Age 15–59 y^f^
**All participants**	28,044	7.2 (6.3–8.1)	NA	NA	NA	NA
**Sex**						
Female	15,826	6.8 (5.9–7.6)*	1	1	1	1
Male	12,218	7.8 (6.5–9.0)	1.2 (1.1–1.3)	1.17 (1.06–1.30)	1.1 (0.8–1.7)	1.4 (1.2–1.6)
**Age**						
1–4 y	289	1.3 (0.0–2.7)	1	1	1	NA
5–9 y	1,360	2.5 (1.9–3.2)	1.6 (0.6–4.1)	1.9 (0.6–5.6)	1.8 (0.6–5.5)	NA
10–14 y	2,324	3.4 (2.4–4.5)	2.4 (1.0–6.0)	2.7 (0.9–7.7)	2.4 (0.8–6.9)	NA
15–19 y	2,520	6.4 (5.1–7.7)*	3.8 (1.5–9.3)	5.7 (2.0–16.5)	NA	1
20–29 y	2,315	8.4 (6.2–10.7)*	5.2 (2.1–12.8)	8.0 (2.8–22.8)	NA	1.3 (1.0–1.7)
30–39 y	4,164	8.3 (6.8–9.7) *	5.0 (2.1–12.3)	7.6 (2.7–21.7)	NA	1.2(0.9–1.6)
40–49 y	6,980	8.7 (7.4–10.0) *	5.7 (2.4–14.0)	7.9 (2.8–22.3)	NA	1.2 (0.9–1.7)
50–59 y	4,076	8.5 (7.4–9.6) *	5.25 (2.2–12.8)	7.5 (2.6–21.1)	NA	1.2 (0.9–1.6)
60–69 y	2,673	6.2 (5.1–7.3) *	3.7 (1.5–9.2)	5.2 (1.8–14.7)	NA	NA
≥70	1,343	5.1 (3.6–6.6) *	3.2 (1.3–8.0)	4.2 (1.4–12.1)	NA	NA
**Ethnicity**						
Han	26,515	7.3(6.3–8.2)	1	1	1	1
Uigur	263	4.7 (3.8–5.6)	0.8 (0.5–1.3)	1.3 (1.1–1.4)	1.1 (0.7–1.8)	1.1 (0.9–1.2)
Tibetan	990	5.8 (4.5–7.1)	0.8 (0.6–1.0)	1.3 (1.1–1.5)	3.6 (1.3–9.6)	1.1 (0.9–1.3)
Others	276	5.2 (2.9–7.5)	0.8 (0.5–1.3)	0.9 (0.7–1.1)	0.0 (0.0–999.9)	0.8 (0.6–1.0)
**Occupation (15**–**59 y)**						
Student	2,629	7.1 (6.0–8.2)	1	NA	NA	1
Farmer	13,285	9.1 (7.5–10.7)	1.3 (1.1–1.6)	NA	NA	0.9 (0.7–1.2)
Worker	960	5.9 (4.3–7.6)	0.8 (0.6–1.2)	NA	NA	0.6 (0.4–0.8)
Cadre	223	4.4 (0.1–8.6)	0.6 (0.3–1.1)	NA	NA	0.5 (0.2–0.9)
Teacher	440	6.4 (3.2–9.5)	0.9 (0.6–1.4)	NA	NA	0.7 (0.5–1.1)
Health care worker	219	4.9 (4.0–5.8)	0.6 (0.3–1.2)	NA	NA	0.6 (0.3–1.2)
Unemployed	1,419	7.6 (6.2–9.0)	1.1 (0.9–1.4)	NA	NA	0.9 (0.7–1.1)
Others^a^	880	8.8 (6.9–10.7)	1.3 (1.0–1.7)	NA	NA	1.0 (0.7–1.2)
**Education (15**–**59 y)**						
Illiterate and primary school	8,125	8.3 (6.8–9.8)	1	NA	NA	1
Middle school	7,239	8.8 (7.3–10.3)	1.0 (0.9–1.1)	NA	NA	1.1 (0.9–1.2)
High school	3,397	8.2 (7.2–9.2)	1.0 (0.8–1.1)	NA	NA	1.1 (0.9–1.3)
Junior college and Undergraduate degree	1,294	5.8 (4.3–7.2)	0.7(0.5–0.9)	NA	NA	0.8 (0.6–1.0)
**Marital status (participants aged >20 y)**						
Never married	957	7.7 (4.8–10.7)	1	NA	NA	1
Married	19,614	8.0 (6.9–9.1)	1.0 (0.8–1.3)	NA	NA	1.1 (0.9–1.4)
Others^b^	579	7.9 (4.8–11.0)	0.9 (0.6–1.3)	NA	NA	1.1 (0.7–1.9)
**Urban/Rural**						
Urban	6,092	6.1 (5.3–7.0)	1	1	1	1
Rural	21,952	7.7 (6.5–8.9)	1.3 (1.1–1.4)	1.5 (1.3–1.7)	1.3 (1.1–1.5)	1.4 (1.1–1.7)
**HBV vaccination**						
No	16,593	8.5 (7.2–9.8)	1	1	1	1
Yes	9,085	5.0 (4.0–5.9) *	0.5 (0.4– 0.6)	0.6 (0.5–0.7)	0.5 (0.3–0.8)	0.5 (0.4–0.6)
Unclear	2,366	7.0 (5.5–8.5)	0.9 (0.8–1.0)	0.8 (0.7–1.0)	0.9 (0.4–1.7)	0.7 (0.5–0.8)
**Household contact with HBV carriers** c						
No	25,454	6.6 (5.6–7.6)	1	1	1	1
Yes	1,690	15.3 (10.5–20.2) *	2.5 (2.2–2.9)	2.6 (2.2–3.0)	3.8 (2.2–6.5)	3.0 (2.5–3.6)
Unclear	900	10.0 (7.6–12.4)	1.5 (1.2–1.9)	1.6 (1.2–2.1)	1.7 (0.8–3.6)	2.1 (1.5–2.9)
**Acupuncture**						
No	25,806	7.11 (6.2–8.0)	1	1	1	1
Yes	2,238	8.1 (6.0–10.1)	1.2 (1.0–1.4)	1.0 (0.8–1.2)	0.4 (0.0–2.8)	1.2 (1.0–1.5)
**Surgery**						
No	19,914	6.9 (6.3–7.5)	1	1	1	1
Yes	8,130	7.9 (5.4–10.2)	1.1 (1.0–1.2)	1.1 (1.0–1.3)	0.8 (0.4–1.5)	1.0 (0.8–1.1)
**Endoscopy examination**						
No	25,820	7.1 (6.2–8.1)	1	1	1	1
Yes	2,224	8.0 (6.4–9.5)	1.2 (1.0–1.4)	1.0 (0.9–1.2)	0.4 (0.1–2.9)	1.1 (0.9–1.3)
**Blood transfusion**						
No	27,034	7.2(6.2–8.1)	1	1	1	1
Yes	1,010	7.5 (4.1–11.0)	1.2 (0.9–1.5)	1.0 (0.8–1.3)	0.4 (0.1–3.0)	0.7 (0.5–1.0)
**Beauty treatments in public places** d						
No	20,670	6.9 (6.3–7.5)	1	1	1	1
Yes	7,374	7.9 (5.3–10.4)	1.1 (1.0–1.201)	1.2 (1.1–1.3)	1.0 (0.6–1.8)	1.2 (1.0–1.4)
Dental treatment						
No	22,343	7.0 (6.2–7.8)	1	1	1	1
Yes	5,701	7.8 (6.1–9.6)	1.1 (1.0–1.3)	1.0 (0.9–1.2)	0.8 (0.3–1.6)	1.1 (1.0–1.3)

HbsAg: hepatitis B surface antigen; NA: not applicable; OR: odds ratio.

*P<0.05 comparing results with the first sub-groups in each variable.

a Mainly includes rural migrant workers and individuals engaging in other temporary work.

b Includes living with partner, divorced, or widowed.

c Includes spouse, parents, brothers, sisters, and other members with HBV infection in the family.

d Includes barber shop shaving, tattooing, and ear piercings.

e Adjusted for sex, age, ethnicity, HBV vaccination, household contact with HBV carriers, acupuncture, surgery, endoscopy, blood transfusion, beauty treatments in public places and dental work.

f Adjusted for sex, age, ethnicity, occupation, education, marital status, HBV vaccination, household contact with HBV carriers, acupuncture, surgery, endoscopy, blood transfusion, beauty treatments in public places, and dental work.

**Table 2:** Prevalence and Determinants of Exposure to Hepatitis B Virus (Positive Anti-HBc) Among Persons ≥1 Year of Age in Wuwei City, China.

Variable	Investigated, n	Prevalence of anti-HBc (95%CI),%	Unadjusted OR (95%CI)	Adjusted OR (95%CI)		
				Age ≥1 y^e^	Age 1–14 y^e^	Age 15–59 y^f^
**All participants**	28,044	43.9 (40.4–47.4)	NA	NA	NA	NA
**Sex**						
Female	15,826	43.9 (40.4–47.4)	1	1	1	1
Male	12,218	43.9 (40.0–47.8)	1.0 (1.0–1.1)	1.2 (1.1––1.3)	1.2 (0.8–1.8)	1.2 (1.1–1.3)
**Age**						
1–4 y	289	18.6 (14.5–22.8)	1	1	1	NA
5–9 y	1,360	14.1 (10.8–17.5)	0.7 (0.5–1.0)	0.8 (0.5–1.1)	1.8 (0.6–5.3)	NA
10–14 y	2,324	15.7 (13.0–18.5)	0.9 (0.7–1.3)	0.9 (0.6–1.2)	2.3 (0.8–6.7)	NA
15–19 y	2,520	23.9 (20.9–26.8)	1.6 (1.2–2.2)	1.4 (1.0–2.0)	NA	1
20–29 y	2,315	36.5 (31.7–41.2) *	2.7 (1.9–3.7)	2.6 (1.9–3.7)	NA	1.6 (1.4–1.9)
30–39 y	4,164	45.8 (41.8–49.8) *	4.0 (2.9–5.5)	3.8 (2.7–5.3)	NA	2.2 (1.9–2.6)
40–49 y	6,980	51.2 (47.1–55.3) *	5.0 (3.6–6.8)	4.6 (3.3–6.5)	NA	2.7 (2.3–3.2)
50–59 y	4,076	55.3 (51.7–59.1) *	6.0 (4.4–8.2)	5.3 (3.8–7.5)	NA	3.1 (2.6–3.7)
60–69 y	2,673	59.3 (55.3–63.3) *	7.0 (5.1–9.6)	6.2 (4.4–8.7)	NA	NA
≥70	1,343	63.1 (59.2–67.1) *	7.4 (5.3–10.2)	6.5 (4.5–9.2)	NA	NA
**Ethnicity**						
Han	26,515	44.2 (40.6–47.8)	1	1	1	1
Uigur	263	43.1 (41.1–45.0)	1.0 (0.8–1.3)	0.9 (0.7–1.1)	1.5 (0.3–7.0)	0.7 (0.5–1.0)
Tibetan	990	24.6 (20.9–28.2) *	0.5 (0.4–0.5)	0.4 (0.4–0.5)	1.0 (0.5–2.4)	0.4 (0.3–0.5)
Others	276	31.5 (24.2–38.9) *	0.7 (0.5–0.9)	0.7 (0.5–1.0)	0.3 (0.0–1.9)	0.7 (0.5–1.0)
**Occupation (15**–**59 y)**						
Student	2,629	25.9 (23.0–28.9)	1	NA	NA	1
Farmer	13,285	48.3 (43.0–53.6) *	2.5 (2.3–2.8)	NA	NA	0.7 (0.6–0.8)
Worker	960	47.2 (41.9–52.6) *	2.6 (2.2–3.0)	NA	NA	0.8 (0.7–1.0)
Cadre	223	50.4 (43.7–57.2) *	2.9 (2.2–3.8)	NA	NA	1.1 (0.8–1.4)
Teacher	440	42.4 (40.0–44.8) *	2.1 (1.7–2.6)	NA	NA	0.9 (0.7–1.1)
Health care worker	219	42.3 (36.8–47.8) *	2.2 (1.6–2.9)	NA	NA	1.0 (0.7–1.3)
Unemployed	1,419	52.1 (50.6–53.6) *	3.1 (2.72–3.6)	NA	NA	1.2 (1.1–1.4)
Others^a^	880	53.9 (50.1–57.7) *	3.3 (2.8–3.9)	NA	NA	1.2 (1.0–1.4)
**Education (15**–**59 y)**						
Illiterate and primary school	8,125	43.2 (38.9–47.6)	1	NA	NA	1
Middle school	7,239	45.4 (41.6–49.3)	1.1 (1.0–1.1)	NA	NA	1.1 (1.0–1.2)
High school	3,397	43. 8 (39.7–47.9)	0.8 (0.8–0.9)	NA	NA	1.1 (1.0–1.2)
Junior college and Undergraduate degree	1,294	42.3 (39.5–45.1)	0.8 (0.7–0.9)	NA	NA	0.9 (0.8–1.1)
**Marital status (participants aged >20 y)**						
Never married	957	35.5 (29.3–41.7)	1	NA	NA	1
Married	19,614	49.6 (45.7–53.5) *	1.7 (1.5–1.9)	NA	NA	1.4 (1.3–1.6)
Others^b^	579	61.3 (55.3–67.4) *	2.5 (2.1–3.1)	NA	NA	1.5 (1.1–1.9)
**Urban/Rural**						
Urban	6,092	47.5 (45.7–49.3)	1	1	1	1
Rural	21,952	42.3 (37.7–46.8)	0.8 (0.7–0.81)	1.0(0.9–1.1)	1.4 (0.7–2.7)	0.9 (0.9–1.0)
**HBV vaccination**						
No	16,593	48.6 (43.7–53. 6)	1	1	1	1
Yes	9,085	33.6 (29.0–38.3) *	0.5 (0.5–0.5)	0.8 (0.8–0.9)	0.5 (0.3–0.7)	0.9 (0.9–1.0)
Unclear	2,366	41.6 (36.2–47.0)	0.8 (0.7–0.8)	0.9 (0.8–1.0)	0.7 (0.3–1.3)	0.9 (0.8–1.0)
**Household contact with HBV carriers** c						
No	25,454	43.0 (39.5–46.5)	1	1	1	1
Yes	1,690	58.2 (54.8–61.6) *	1.7 (1.6–1.9)	1.8 (1.6–2.0)	5.4 (3.1–9.4)	1.9 (1.7–2.2)
Unclear	900	43.0 (33.9–52.2)	0.9 (0.7–1.0)	1.3 (1.1–1.5)	2.1 (1.0–4.6)	1.3 (1.1–1.6)
**Acupuncture**						
No	25,806	42.7 (39.2–46.2)	1	1	1	1
Yes	2,238	57.5 (52.3–62.6) *	1.8 (1.6–1.9)	1.1 (1.0–1.2)	0.9 (0.3–2.7)	1.1 (1.0–1.2)
**Surgery**						
No	19,914	40.3 (36.5–44.2)	1	1	1	1
Yes	8,130	51.4 (48.6–54.2) *	1.5 (1.5–1.6)	1.2 (1.1–1.3)	0.7 (0.4–1.3)	1.2 (1.1–1.3)
**Endoscopy examination**						
No	25,820	42.6(39.1–46.1)	1	1	1	1
Yes	2,224	58.1 (55.2–61.0) *	1.8 (1.7–2.0)	1.2 (1.0–1.3)	0.5 (0.2–1.5)	1.2 (1.0–1.3)
**Blood transfusion**						
No	27,034	43.4(39.8–47.0)	1	1	1	1
Yes	1,010	56.1 (48.7–63.4) *	1.7 (1.5–1.9)	1.0(0. 9–1.2)	0.7 (0.2–2.4)	1.0 (0.9–1.2)
**Beauty treatments in public places** d						
No	20,670	41.8 (37.8–45.7)	1	1	1	1
Yes	7,374	49.1 (45.3–53.0)	1.3 (1.2–1.3)	1.1 (1.0–1.2)	1.4 (0.8–2.5)	1.1 (1.0–1.2)
**Dental treatment**						
No	22,343	41.6(37.9–45.3)	1	1	1	1
Yes	5,701	53.1 (50.7–55.5) *	1.7 (1.6–1.8)	1.0 (1.0–1.1)	0.9 (0.4–2.0)	1.0 (0.9–1.1)

Anti-HBc: hepatitis B core antigen; NA: not applicable; OR: odds ratio.

*P<0.05 comparing results with the first sub-groups in each variable.

a Mainly includes rural migrant worker and individuals engaging in other temporary work.

b Includes living with partner, divorced, widowed.

c Includes spouse, parents, brothers, sisters, and other family members with HBV infections.

d Includes barber shop shaving, tattooing, and ear piercing.

e Adjusted for sex, age, ethnicity, HBV vaccination status, household contacts with HBV infection, acupuncture, surgery, endoscopy, blood transfusion, beauty treatments in public places and dental work.

f Adjusted for sex, age, ethnicity, occupation, education, marital status, HBV vaccination, household contacts with HBV infection, acupuncture, surgery, endoscopy, blood transfusion, beauty treatments in public places, and dental work.

**Table 3:** Prevalence and Determinants Associated with the Presence of Anti-HBs and Without Anti-HBc Among Persons Aged ≥1 Year of Age in Wuwei City, China.

Variable	Investigated, n	Prevalence of Positive Anti-HBs and Negative Anti-HBc (95% CI), %	Unadjusted OR (95%CI)	Adjusted OR (95%CI)		
				Age ≥1 y^c^	Aged 1–14 y^c^	Aged 15–59 y^d^
**All participants**	28,044	23.5 (21.6–25.3)	NA	NA	NA	NA
**Sex**						
Female	15,826	22.8 (20.5–25.2)	1	1	1	1
Male	12,218	24.4 (22.4–26.3)	1.1(1.1–1.2)	0.93 (0.88–1.00)	1.1 (1.1–1.3)	0.9 (0.8–1.0)
**Age**						
1–4 y	289	37.7 (30.4–45.0)	1	1	1	NA
5–9 y	1,360	41.7 (33.8–49.5)	1.1 (0.8–1.4)	1.2 (0.9–1.6)	1.2 (0.9–1.6)	NA
10–14 y	2,324	44.3 (37.7–50.8)	1.1 (0.9–1.5)	1.3 (1.0–1.7)	1.3 (1.0–1.7)	NA
15–19 y	2,520	44.8 (41.2–48.4)	1.2 (1.0–1.6)	1.1 (0.9–1.5)	NA	1
20–29 y	2,315	28.3 (23.4–33.1)	0.6 (0.5–0.8)	0.6 (0.4–0.8)	NA	0.8(0.7–0.9)
30–39 y	4,164	19.4 (16.1–22.7)*	0.4 (0.3–0.5)	0.4 (0.3–0.5)	NA	0.7(0.6–0.8)
40–49 y	6,980	16.6 (14.1–19.0) *	0.3 (0.2–0.4)	0.3 (0.3–0.5)	NA	0.7(0.6–0.8)
50–59 y	4,076	15.3 (13.7–16.9) *	0.3 (0.2–0.4)	0.3 (0.3–0.4)	NA	0.8(0.6–0.9)
60–69 y	2,673	14.9 (12.3–17.6) *	0.3 (0.2–0.4)	0.3 (0.3–0.5)	NA	NA
≥70	1,343	14.7 (12.0–17.3) *	0.3 (0.2–0.4)	0.4 (0.3–0.5)	NA	NA
**Ethnicity**						
Han	26,515	23.5 (21.6–25.5)	1	1	1	1
Uigur	263	20.7 (19.0–22.4)	0.9 (0.7–1.2)	0.9 (0.7–1.2)	1.0 (0.5–1.8)	0.8 (0.5–1.2)
Tibetan	990	24.91 (16.6–33.3)	1.1 (0.9–1.3)	1.4 (1.1–1.7)	0.8 (0.5–1.1)	1.7 (1.3–2.1)
Others	276	19.06 (6.8–31.3)	0.6 (0.5–0.9)	0.8 (0.5–1.2)	0.6 (0.3–1.1)	0.8 (0.5–1.4)
**Occupation (15**–**59 y)**						
Student	2,629	43.7 (40.1–47.2)	1	NA	NA	1
Farmer	13,285	16.4 (14.9–18.0) *	0.3 (0.2–0.3)	NA	NA	0.5 (0.5–0.6)
Worker	960	19.2 (18.6–19.7) *	0.3 (0.3–0.4)	NA	NA	0.6 (0.5–0.7)
Cadre	223	26.4 (23.2–29.7) *	0.5 (0.4–0.7)	NA	NA	0.8(0.5–1.1)
Teacher	440	33.8 (31.3–36.4) *	0.7 (0.5–0.8)	NA	NA	1.1 (0.8–1.3)
Health care worker	219	43.9(40.8–46.9)	1.0 (0.7–1.3)	NA	NA	1.4 (1.0– 1. 9)
Unemployed	1,419	20.3(20.0–20.7) *	0.3(0.3–0.4)	NA	NA	0.6 (0.5–0.7)
Others^a^	880	20.0 (16.5–23. 5) *	0.3 (0.3–0.4)	NA	NA	0.6 (0.5–0.8)
**Education (15**–**59 y)**						
Illiterate and primary school	8,125	16.5 (14.7–18.4)	1	NA	NA	1
Middle school	7,239	21.5 (19.5–23.5) *	1.5 (1.4–1.6)	NA	NA	1.0 (0.9–1.1)
High school	3,397	26.2 (22.8–29.7) *	1.9 (1.7–2.1)	NA	NA	1.0 (0.9–1.2)
Junior college and Undergraduate degree	1,294	34.2 (30.2–38.2) *	2.8 (2.5–3.2)	NA	NA	1.7 (1.5–2.0)
**Marital status (participants aged >20 y)**						
Never married	957	30.8 (24.3–37.3)	1	NA	NA	1
Married	19,614	17.0 (14.8–19.3) *	0.5 (0.4–0.5)	NA	NA	0.6 (0.5–0.6)
Others^b^	579	13.9 (10.5–17.4) *	0.4 (0.3–0.5)	NA	NA	0.75 (0.55–1.02)
**Urban/Rural**						
Urban	6,092	24.0 (19.7–28.4)	1	1	1	1
Rural	21,952	23.3 (21.5–25.0)	0.9 (0.9–1.0)	1.0(0.9–1.1)	1.2 (1.0–1.5)	0.91 (0.82–1.00)
**HBV vaccination**						
No	16,593	16.5 (14.2–18.8)	1	1	1	1
Yes	9,085	38.8 (35.4–42.2) *	3.3 (3.1–3.5)	2.4 (2.2–2. 6)	1.7 (1.5–2.0)	2.7 (2.5–2.9)
Unclear	2,366	26.0 (21.2–30.8) *	1.7 (1.5–1.9)	1.6 (1.4–1.7)	1.2 (0.9–1.4)	1.7 (1.5–1.9)

Anti-HBs: antibodies against HBV surface antigen; anti-HBc: hepatitis B core antigen; NA: not applicable; OR: odds ratio.

*P<0.05 comparing results with the first sub-groups in each variable.

a Mainly includes rural migrant worker and individuals engaging in other temporary jobs.

b Includes living with partner, divorced, widowed.

c Adjusted for sex, age, ethnicity, and HBV vaccination.

d Adjusted for sex, age, ethnicity, occupation, education, marital status and HBV vaccination.

### 2. Prevalence of chronic HBV infection and exposure characteristics

The prevalence of chronic HBV infection in children and adolescents were 1.3% (aged 1–4 year age group), 2.5% (5–9 year age group), and 3.4% (10–14 year age group), respectively. Prevalence increased with age, peaking in the 40–49 year old age group (8.7%), and decreasing in the 50–59 (8.5%), 60–69 (6.2%), and the ≥70 (5.10%) year-old age groups (Table 1). HBV infection was more common in men (7.9%) than in women (6.9%). Cadre was the predecessor of the civil servants/public officers, they had to pass medical examination before they were hired, the HBsAg positive one was not to be hired before 2010. Cadre had the lowest prevalence rate (4.4%) followed by health care workers (4.6%), and farmers had the highest prevalence (9.1%). Prevalence rates based on ethnicity were as follows: individuals of Han decent had the highest HBsAg prevalence (7.3%), followed by individuals of Tibetan decent (5.8%), other minority ethnic groups (5.2%), and last individuals of Uigur decent (4.7%). Among persons aged 15–59 years, the lowest prevalence was in the junior college and undergraduate degree population (5.8%). Unvaccinated individuals had higher prevalence rates of HBsAg (8.5%) compared to levels observed in HBV vaccinated individuals (5.0%, OR = 0.6, CI: 0.5–0.7). Persons who had household contact with HBV carriers (OR = 2.6, CI: 2.2–3.0) had the highest HBsAg prevalence rate (15.3%) among all subgroups examined. Having household contact with HBV carriers (OR = 3.8, 95% CI:2.2–6.5) and lack of hepatitis vaccination (OR = 2.0, 95% CI: 1.4–3.3) were the risk factors for HBV infection in children aged 1–14 years.

### 3. Prevalence of HBV exposure based on age

Prevalence of exposure to HBV increased with age from 14.1% among persons 5–9 years of age to 63.1% among persons >69 years of age (Table 2). Exposure to HBV was similar between men (43.9%) and women (43.9%), but more common in individuals of Han (44.2%) and Uigur (43.1%) decent than in persons of Tibetan (24.6%) decent or of other ethnicities (31.5%) (Table 2). The prevalence was higher in persons who were living with partners, were divorced, widowed and had household contact with HBV carriers. High HBV exposure rates were observed in rural migrant workers or other temporarily employed individuals (53.9%) having a history of acupuncture, surgery, endoscopic examination, beauty treatments in public places, and having received dental work.

### 4. Prevalence of vaccine-induced immunity

Among children aged 1-year old (n = 144), 66.6% (CI, 39.7–93.4%) had positive serum anti-HBs responses, whereas children aged 2, 3, and 4 years of age the rates were 54.22% (CI, 20.2–68.2%), 49.3% (CI, 34.8–63.7%), and 39.3% (CI, 32.6–46.1%), respectively. There were no significant differences observed in the prevalence rates of anti-HBs antibodies based on gender, age, ethnicity, or marital status. The prevalence of positive anti-HBs responses combined with negative anti-HBc results was suggestive of the persistent presence of protective antibodies after vaccination in 23.5% of participants (Table 3). Prevalence of vaccine-induced immunity negatively correlated with age, ranging from 44.8% among persons aged 15–19 years to 15.6% among persons >69 years of age (Table 3). Individuals with the highest levels of vaccine-induced immunity had an HBV vaccination history (OR = 2.7, CI: 2.5–2.9), completed junior college or an undergraduate degree (OR = 1.7, CI: 1.5–2.0), or were health care workers (OR = 1.4, CI: 1.0–1.9).

## Discussion

In northwest China, hepatitis B infections represent one of the most important threats to public health accounting for approximately 43% of notifiable infections yearly[16]. In this study, the prevalence of HBsAg was 7.2% in the general population and 2.3% of the 1–9 year olds had chronic HBV infections. The HBV infection rates in Wuwei City remain a serious concern even after the implementation of the routine HBV immunization program established in 2002 and the HBV catch-up vaccination program for children and adolescents established in 2009.

The prevalence of chronic HBV infection in Wuwei City in 2010 was 7.4% among persons 1–59 years of age, which is slightly higher than previous national survey results obtained in 2006 (7.2%). However, the prevalence of HBsAg among children 5–9 years of age in Wuwei City was significantly higher than rates reported from a national survey in 2006 for children of a similar age, possibly due to the lower vaccine coverage rates at birth between 2001–2005 in western China than in eastern China[6], [17], which resulted in increased HBV infection risks[7], [18]. Having household contact with HBV carriers has been considered to be important risk factors for hepatitis B infection, which included mother-to-child transmission and other members transmission. The OR value of household contact with HBV carriers was 2.6 for the all the participants, 3.8 for 1–14 years of age and 3.0 for the 15–59 years of age, which suggested that this risk factor was more important for children than adults, the OR value was similar with the study of acute hepatitis B in Shanghai[15]. A recent study in Wuwei City indicated that the rate of mother-to-child transmission of HBV was still high (7.3%) and that the absence of HBV vaccination after birth was a possible independent risk factor for increased rates of mother-to-child HBV transmission[19]. Lack of HBV vaccination was the risk factor of HBV chronic infection and exposure to HBV for all the participants. Beauty treatments, which included barber shop shaving, tattooing and ear piercing, were common in western China. In this study, 26.3% (7,374/28,044) of the general population had the history of beauty treatments, though these public places lacked of effective health surveillance compared with medical units[20].

Exposure to HBV (past or chronic infections) in the Wuwei population was 7 times more common than other chronic infections, consistent with the fact that most individuals exposed to HBV in adulthood are able to “clear” the virus without persistence of HBsAg in serum. The prevalence of HBV exposure was 41.2% among persons 1–59 years of age and was significantly higher than rates described in the national survey in 2006 (34.1%), especially for children 1–14 years of age. These observations suggested that Wuwei City was endemic for HBV with prevalence rates greater than the national average. The prevalence of anti-HBc antibodies among children 1–4 years of age was higher than children 5–9 years of age. The higher prevalence of anti-HBc antibodies in children may partly be due to the immunization status of their mothers since the prevalence of anti-HBc positive mothers (43.1%, CI: 39.3–46.8%) was significantly higher than the prevalence of anti-HBc negative mothers (30.9%, CI: 27.6–34.1%) which decreased with age[21]. The OR value of household contact with HBV carriers for exposure to HBV was 1.8 for the all the participants, 5.4 for 1–14 years of age and 1.9 for the 15–59 years of age, which showed that household contact was more important for children than adults. Invasive medical procedure (acupuncture, surgery and endoscopy examination) and blood transfusion were also the risk factors for expose to HBV, which suggested the medical factors were also played an important role for the expose to HBV and health surveillance to the medical units should be strengthened.The prevalence of anti-HBs was 49.5% among persons 1–59 years of age which was slightly lower than rates reported in the national survey of 2006 (50.1%). However, the prevalence rates among children 1–4 years of age was significantly lower than rates reported in the national survey of 2006[3]. The prevalence of anti-HBs sharply declined from 66.5% in 1-year olds to 39.3% in 4-year olds and a significant decrease in serum anti-HBs observed within the first 3 years after vaccination may have been due to administration of low vaccine dosages. A recent large population study in China showed that the antibody response rate following administration of 10 µg/dose hepatitis B vaccine was higher than the response elicited by the 5 µg/dose administered to newborns, suggesting that raising the HBV vaccine dose may be necessary in infants born in endemic areas[22]. The prevalence among children 15–19 years old was significantly higher than the national average reported in 2006 due to catch-up vaccinations administered to individuals 14–15 years old and individuals 15–24 years old attending junior colleges during 2009–2010.

The differences in prevalence rates among individuals presenting with vaccine-induced immunity gradually disappeared between rural and urban areas, and in different regions among persons <20 years of age as the HBV vaccination cost diminished to no charge for newborns and other select populations. Although the prevalence of chronic infections and HBV exposure in Wuwei City was 27 and 9 times higher, respectively, than rates observed in the United States between 1999–2006, the prevalence of vaccine-induce immunity in Wuwei City was slightly higher than United States rates between 1999–2006[4], [23] suggesting that the comprehensive HBV vaccination was successful. However, the prevalence of vaccine-induce immunity in Wuwei City was significantly lower than rates reported in United States between 1999–2006 among 13–17 year-olds because about 15% of individuals in this age group acquired immunity as a result of infection. Among individuals >20 years of age, the majority (62.3%) presenting with anti-HBs antibodies acquired immunity following exposure to HBV.

Before the HBV vaccine was widely available in China, HBV infections were acquired rapidly either perinatally or transmitted between children 1–4 years of age[24]. After routine implementation of the HBV immunization program in 2002 the primary obstacle to overcome was vaccine administration to children of lower socioeconomic levels, which hampered the efficacy of the vaccination program in infants since some parents did not precisely follow the immunization schedules, especially at younger ages due to low socio-economic status and residence in remote areas that limited access to health and educational services[25], [26]. Besides, the delayed administration of the first HBV vaccine dose to premature infants was a significant risk factor for HBV mother-to-child transmission in individuals living in Wuwei City[19]. Because a high proportion of women of childbearing age (15–49 years of age) remain HBsAg positive (7.2%, CI: 6.0–8.5%) the national program must continue to improve immunization practices if higher levels of vaccination are to be reached. Specifically, timely administration of the HBV vaccine combined with hepatitis B immune globulin need to be administered to at risk individuals[27].

After implementing the universal HBV immunization policy for all newborns, in addition to the catch-up programs for children and adolescents[28], protection of susceptible adults has become more important than ever in China. Sexual contact with chronically infected persons has been recognized as one of the most efficient routes of HBV transmission[29]. Data from this study showed high prevalence rates of HBsAg among sexually active persons from adolescence to mid-adulthood, strongly suggesting that sexual transmission of HBV represents a major infection route after disposable syringe programs were implemented, in addition to the implementation of safe blood transfusion practices[30]. Therefore, the coverage of the current HBV vaccination program needs to be expanded to include sexually active young adults in China. Considering the high mobility of the population and the nature of the vaccine immune response in this group, the traditional 5 µg/dose, 0–1–6 schedule used for immunizing infants is not suitable for adults living in an endemic area. To explore cost effectiveness, vaccination schedules designed to expand the vaccination coverage and duration of protection in adults is a priority[31], [32].

The present study has some limitations. First, the study design did not account for occult infections, that is, individuals seronegative for HBsAg and acute infections that would preclude detection of anti-HBc antibodies[33]. Second, some selection bias may have occurred since 31.1% of potential participants were excluded before the interview since they worked outside of the city as rural migrant workers (in most cases), and this was most true status in western China. This could have caused an underestimation in the prevalence of HBsAg since most of these individuals presented with high levels of HBsAg (primarily male farmers, 16–40 years of age). This highly mobile group represents both victims and carriers of HBV infections. This is a growing population in western China which should be the target of prevention strategies[34]. Third, the risk factors associated with chronic HBV infections were obtained from a cross-sectional survey and should be verified using new HBV infection surveys because some risk factors (including the vaccination history for adults) occurred at a long time ago and was obtained based on memory, recall bias may exist. For these reasons, the prevalence of HBV infection is likely to be underestimated.

In conclusion, this population-based study in western China provided information that will be useful in planning preventive strategies. Although vaccination against HBV infection has become a standard practice, we found that the prevalence of HBV was as high as 7.2%. HBV infections will continue to remain a serious public health problem in western China in the coming decades. Hepatitis B vaccine immunization strategies therefore need to be improved further and designed to include coverage of highly mobile adult populations.
